# Impact of intermittent fasting on physical activity: a national survey of Chinese residents aged 18–80 years

**DOI:** 10.3389/fphys.2025.1582036

**Published:** 2025-05-12

**Authors:** Feiying He, Shiyu Bai, Xiangchun Xu, Jingqiao Miao, Hongwen Yu, Jiale Qiu, Yibo Wu, Yangdong Fan, Lei Shi

**Affiliations:** ^1^ School of Health Management, Southern Medical University, Guangzhou, Guangdong, China; ^2^ School of Basic Medical Sciences, Southern Medical University, Guangzhou, Guangdong, China; ^3^ Guangdong Provincial People’s Hospital (Guangdong Academy of Medical Sciences), Southern Medical University, Guangzhou, Guangdong, China; ^4^ School of Public Health, Southern Medical University, Guangzhou, Guangdong, China; ^5^ School of Stomatology, Southern Medical University, Guangzhou, Guangdong, China; ^6^ School of Public Health, Peking University, Beijing, China; ^7^ School of Health Management, Guangzhou Medical University, Guangzhou, Guangdong, China

**Keywords:** intermittent fasting, physical activity, multiple logistic regression, health management, China

## Abstract

**Objectives:**

This study aims to investigate the prevalence of intermittent fasting (IF) among Chinese residents aged 18–80 and assess its impact on physical activity (PA) levels.

**Methods:**

Data were sourced from the Psychology and Behavior Investigation of Chinese Residents, a nationally representative cross-sectional survey conducted between June 20 and 31 August 2022. A multistage stratified cluster sampling method was used. Propensity score matching (PSM) was applied to compare PA levels between individuals practicing IF and those not practicing it. Multiple logistic regression and subgroup analysis were performed to explore associations between PA levels and relevant factors.

**Results:**

IF was practiced by 9.78% of participants, with the highest prevalence (70.78%) among those aged 18–34. While there were no significant differences in baseline characteristics between the IF and non-IF groups, sleep duration differed. IF was significantly associated with reduced PA levels (OR = 0.769, 95%CI: 0.657–0.900), and subgroup analysis highlighted the effect of sleep patterns on PA.

**Conclusion:**

IF is common among younger Chinese residents and correlates with lower PA levels, indicating a potential need for individualized health guidance to balance dietary strategies with PA.

## Introduction

Intermittent fasting (IF) has been proven to be an effective and healthy pattern (Mattson and de Cabo, 2019). IF involves consuming very few or no calories during a specific period of time. There are two main types of fasting strategies: limiting food intake by reducing the amount of food per meal or only eating within the daily time limit; and IF, which involves alternating periods of fasting and feeding. An example of IF is the 5:2 method, where individuals sit a reduced portion of a meal 2 days a week. Numerous studies have rediscovered the benefits of IF (Mattson and de Cabo, 2019; Liu et al., 2017), enhancing metabolic activity (Vasim et al., 2022), improving blood cholesterol and blood pressure (Li et al., 2021), and preventing diseases like cancer, heart disease, and nerve damage (Jamshed et al., 2022; Salvadori et al., 2021; Borowski and Vuong, 2022).

The rate of unhealthy eating and the prevalence of obesity have gradually increased along with people’s living standards (Caballero, 2019). Simultaneously, there has been an increase in health awareness and the desire for body management, driven by the widespread popularity of social media and access to information. There is a strong correlation between weight loss and dietary behaviour (Aparicio-Martinez et al., 2019). IF can be widely adopted in residents’ daily lives as a simple and straightforward and dietary strategy, without the need to consider complicated elements such as food calories or nutritional combinations (Freire, 2020). According to the research by Longo and Mattson (Longo and Mattson, 2014), IF can improve conditions such as asthma and depression through mechanisms including ketogenesis. In addition, Stekovic et al. (2019) found that reduced levels slCAM-1 (an age-associated inflammatory marker), low-density lipoprotein, and the metabolic regulator triiodothyronine after long-term alternate day fasting. Although abundant studies have demonstrated the positive effects of IF on health outcomes and prevent disease (Longo and Mattson, 2014; Stekovic et al., 2019; Zhang et al., 2020), it is important to acknowledge that there are also evidences suggesting potentially harmful effects of IF. IF may reduce muscle strength in the limbs (Lowe et al., 2020), impair limb function (Estrada-deLeon et al., 2021), and decrease insulin, thyroid hormone, and testosterone levels (Kim et al., 2021; Cienfuegos et al., 2022), all of which are associated with physical activity (PA). Moreover, IF has been associated with eating disorders and psychiatric issues related to psychological behaviour (Ganson et al., 2022).

IF has a long history and is deeply connected to culture and religion. It is often seen as a therapeutic treatment (Visioli et al., 2022). In recent years, IF has gained popularity particularly among those who are seeking to lose weight (Varady et al., 2022), and is widely adopted as a dietary strategy among overweight individuals (Freire, 2020). However, there is limited information available on the prevalence of IF among residents in existing literature. A study conducted on Canadian adolescents and young adults found that within the past 12 months, 47.7% of women, 38.4% of men, and 52.0% of transgender or gender-uncertain people reported practising IF (Ganson et al., 2022).

PA is typically defined as “any physical activity that involves the contraction of skeletal muscle” and results in energy expenditure (Caspersen et al., 1985). This includes various sports like running, swimming, and ball games, as well as everyday activities like walking and cycling. The components of PA, according to the International Physical Activity Scale (IPAQ), include frequency, duration, and intensity. The physical effort involved in participating in PA can be analyzed through its constituent elements, which include: 1) Frequency, referring to the amount of PA performed within a designated time period; 2) Duration, indicating the length of time spent on PA (Shi et al., 2022); and 3) Intensity.

According to a study conducted on university students in Northeast China, 13.3%, 39.8%, and 47.2% of individuals had high, medium, and low PA levels, respectively (Ge et al., 2019). Another study on middle-aged and older adults in China, found that 30.3%, 24.4%, and 45.3% of participants had high, medium, and low PA levels, respectively (He et al., 2021). Engaging in high levels of PA can lower the risk of depression and cognitive function decay (Choi et al., 2019; Voss et al., 2019), type 2 diabetes, obesity, and cardiovascular disease (Ling and Ronn, 2019; Pan et al., 2021). PA is also associated with physical literacy and socioeconomic factors (Caldwell et al., 2020; Kyan and Takakura, 2022). Both IF and PA are popular strategies for weight loss. However, the impact of IF on PA is relatively complex. Some studies have found that IF can reduce body fat content and improve physical function (Martinez-Rodriguez et al., 2021), while others have found no significant improvement in physical performance (Levy and Chu, 2019). Additionally, IF may have negative effects, such as muscle loss, which can be disadvantageous to PA (Martinez-Rodriguez et al., 2021). Therefore, it is necessary to further explore the relationship between IF and PA.

IF may affect sleep quality, which in turn affects PA levels. There has been a research report that IF caused by changes in eating time may lead to shorter sleep time and poor sleep quality (Yamaguchi et al., 2013), which may lead to insulin resistance and increase the risk of diabetes and cardiovascular diseases. However, there is currently little evidence to demonstrate a relationship between IF and sleep patterns. The timing of meals in IF may also impact circadian rhythms, which are crucial for regulating sleep patterns. Altering meal times can shift the phase of circadian rhythms, potentially delaying the onset of sleep and disrupting sleep architecture. Sleep quality directly influences PA levels; adequate sleep can enhance reaction times, alleviate fatigue, and improve physical performance (Souissi et al., 2020). Conversely, insufficient sleep may decrease the likelihood of engaging in active physical activities and increase the risk of sports injuries (Bromley et al., 2012). Therefore, understanding the effects of IF on both sleep and PA is essential for maximizing the health benefits of IF. Although IF can promote health and aid in weight management, the timing of its implementation must be carefully considered to minimize negative impacts on sleep. Ensuring good sleep hygiene and regular PA can amplify the health advantages of IF while mitigating potential risks.

This study aims to address these gaps by examining the prevalence of IF behaviour among Chinese residents and analyzing the relationship between IF behaviour and PA intensity. The hypothesis of this study suggests that there will be a significant difference in PA intensity among residents with different IF behaviors.

## Methods

### Study design and participants

Data for this study were collected from the Psychology and Behaviour Investigation of Chinese Residents (PBICR), a cross-sectional, nationally representative survey conducted in China. The survey was carried out between 20 June and 31 August 2022 using a multistage stratified cluster sampling method. A total of 148 cities, 202 counties, and 309 villages/towns were included in the PBICR, covering 23 provinces, four municipalities, and five autonomous regions in mainland China (excluding Taiwan, Hong Kong, and the Macaw Special Administrative Region) (Zhang et al., 2025; Yang et al., 2024). The clusters were sampled based on equal probability, while individuals were sampled within each cluster. For each city, at least one investigator or survey team was recruited. If a respondent had the ability to think but lacked the physical capability to fill out the questionnaire, the investigator conducted a one-on-one interview and recorded the answers on their behalf. The inclusion criteria for residents in this study were as follows: 1) being permanent Chinese resident who had not left mainland China for more than 1 month within a year; 2) providing written informed consent; and 3) volunteering to participate. Residents who were unconscious, mentally abnormal, or had cognitive failures were excluded from the study. Ultimately, a total of 30,503 questionnaires that met the ethical examination guidelines and represented the national population were obtained.

The sample screening process resulted in the selection of 25,679 valid questionnaires from the initial pool of 30,503 questionnaires, which involved screening based on age, body mass index (BMI) range and outliers. The recovery rate of valid questionnaires was 91.57%. From the selected valid questionnaire, participants aged between 18 and 80 years and with a BMI ranging from 15.8 to 43.6 were included. For the primary outcome analysis, Propensity Score Matching (PSM)-matched data were used, consisting of two groups: the IF group and non-IF group each comprising 2,512 participants.

### Measurement

#### Self-compiled general situation questionnaire

A self-compiled general situation questionnaire was used to assess the socio-demographic characteristics of the residents. The questionnaire included various demographic indicators, such as age (18–34 years old, 35–44 years old, 45–59 years old, and 60–80 years old), gender (male and female), BMI (below 18.5, 18.5–23.9, 24–27.9, and over 28), educational attainment (high school or below, undergraduate or junior college, and master’s or above), income level (below 2,000; 2,001–4,000; 4,001–6,000; 6,001–12,000; over 12,001), smoking (no, quit, and yes), alcohol usage (never, recently started, in the past, all the time), and sleep time (below 5 h, 5–6 h, 6–7 h, over 7 h).

#### Intermittent fasting behavior

In this study, the participants’ IF behaviour was assessed using one item which was adapted from the previous study (Varady et al., 2022). The item asked, “Have you practiced intermittent fasting in the past year?” with response options of “Yes” and “No.” This question, used to measure whether the participants have IF bahaviour, has demonstrated good reliability in previous studies. The item was administered face-to-face by trained investigators using standardized procedures. Examples of IF patterns were provided to reduce ambiguity and recall bias.

#### International physical activity scale (IPAQ-7)

**FIGURE 1:**
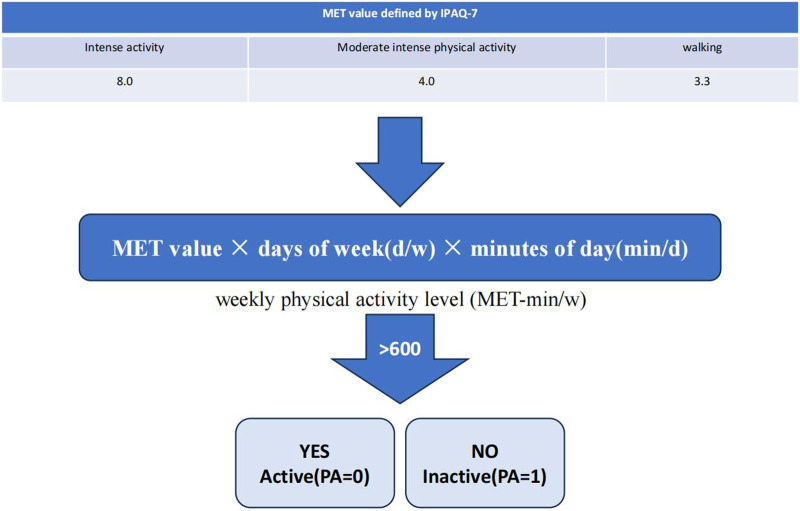
Two levels of physical activity.

The International Physical Activity Scale, also known as the International Physical Activity Questionnaire (IPAQ), was developed as a global tool for measuring physical activity, which has demonstrated acceptable reliability (Cronbach’s α = 0.80 in our sample) (Cleland et al., 2018; Puciato et al., 2017). Reliability and validity studies conducted in 2000 across 14 sites in 12 countries indicated that it had an acceptable valuation for usage. For this study, the short IPAQ questionnaire was utilised, consisting of seven single-item questions that cover three types of activities: intense physical activity, (moderate intensity physical activity, and walking. These questions aimed to gather information on recent physical activity levels. For instance, one question asked “In the last 7 days, how many days did you participate in activities such as running, ball games, fast cycling, and other intensive activities?” Participants could choose from eight possible options, ranging from “no intensive activity” and “1 day” to “7 days”. The Metabolic Equivalent (MET value, for intense activity, moderate intense physical activity and walking are 8.0, 4.0 and 3.3, respectively) × days of week (d/w) × minutes of day (min/d) equals to weekly physical activity level (MET-min/w) (the sum of three intensity physical activity). According to the World Health Organization’s recommendations for physical activity, adults aged 18 and over should engage in either: 1) at least 150–300 min of moderate-intensity aerobic physical activity per week, 2) at least 75–150 min of vigorous-intensity aerobic physical activity, or 3) an equivalent combination of moderate and vigorous-intensity activity. We have categorized participants’ levels of physical activity into two groups: active (PA = 0) and inactive (PA = 1) (Figure 1). The Cronbach’s alpha coefficient of IPAQ in this study was 0.80 (Cleland et al., 2018; Craig et al., 2003).

### Statistical analysis

The data for this investigation analysed using R 4.4.0 software. PSM was employed in this observational study design to recreate randomized controlled trials. PSM simplify the causal analysis of observational data by balancing covariates between treatment groups, enhancing inference by mimicking randomization, thereby allowing for more robust and flexible statistical analyses (Peter, 2011). To compare the differences between residents with and without IF based on individual baseline characteristics, the Pearson chi-square test was used for categorical variables, and the one-way test was used for all non-parametric continuous variables. Age, gender, BMI, educational attainment, income level, smoking, alcohol usage, and sleep duration were the eight factors considered in the PSM analysis, to control for potential confounding effects. To achieve an optimal match, a caliper value of 0.001 was set, and the 1:1 nearest neighbour matching method was applied to calculate the trend score. A standardised difference of 0.1 or below indicated a negligible difference between the two groups. The caliper value of 0.001 was selected based on the following considerations: Pre-matching analyses revealed substantial imbalances in key covariates (such as sleep duration, SMD = 0.15; age distribution, SMD = 0.12). In pilot analyses, wider calipers resulted in >30% of covariates exceeding the SMD threshold of 0.1, violating the balance requirement (Varady et al., 2022). With caliper = 0.001, all covariates achieved SMD <0.05, ensuring minimal residual confounding. This stringent caliper aligns with prior studies examining dietary-behavioral interactions, where precise matching was critical to isolate intervention effects (Peter, 2011). Statistical significance was set at *p* < 0.05. significance levels were further classified as follows: *p* < 0.05, *p* < 0.01, *p* < 0.001.

To determine the difference in PA intensity between the two groups, the frequency (n) and proportion percentage (%) were analysed individually. PSM was employed to minimize the effect of confounding factors on PA intensity and to better explore other risk factors associated with PA intensity. Logistic regression was used when multiple variables were considered simultaneously. Multivariate logistic regression balances confounding factors by simultaneously analyzing the relationship between multiple independent variables and the outcome, adjusting for the influence of other variables to isolate the effect of each predictor (Jia et al., 2021). Multivariate logistic regression models were performed to compute the relationship between physical activity, gender, age and so on. A mediation analysis was conducted to discuss the potential effects of sleep duration to PA. And we also conducted subgroup analysis to stratify the relevance between sleep time and physical activity. A 0.05 threshold for statistical significance was used. 95% confidence intervals (CIs) and odds ratios (ORs) were used to display the results.

## Results

### Baseline characteristics after matching

PSM method is utilized to address and eliminate selection bias in this study (Shipman et al., 2017). The 2,512 participants in the groups with and without IF showed no statistically significant differences in the observed variables after PSM (*p* > 0.05, Table 1). A notable difference was observed in sleep duration; the IF group tended to sleep less than 5 h or more than 7 h compared to the non-IF group, which is statistically significant (*p* = 0.038). Out of the total 5,024 participants, 3,560 (70.9%) were aged 18–34; 3,248 (64.6%) were mainly female; 3,172 (63.1%) had a healthy BMI, which ranged from 18.5 to 23.9; 1,432 (28.5%) had incomes between 2,001 and 4,000 yuan; 4,087 (81.3%) reported never smoking; over half of the participants 2,971 (59.1%) reported never drinking; and 1,821 participants (36.2%) reported sleeping for 5–6 h each night.

**TABLE 1:** Baseline characteristics of the post-propensity score matching between the non-intermittent fasting and intermittent fasting groups (*N* = 5024).

Variables	IF		p	SMD
	No (%), n = 2512	Yes (%), n = 2512		
Age (years)				
18–34	1782 (70.9)	1778 (70.8)	0.900	0.022
35–44	326 (13.0)	314 (12.5)		
45–59	318 (12.7)	328 (13.1)		
60–80	86 (3.4)	92 (3.7)		
Gender				
Male	896 (35.7)	880 (35.0)	0.658	0.013
Female	1616 (64.3)	1632 (65.0)		
BMI (kg/m^2^)				
<18.5	271 (10.8)	298 (11.9)	0.651	0.036
18.5–23.9	1589 (63.3)	1583 (63.0)		
24.0–27.9	496 (19.7)	480 (19.1)		
≥28.0	156 (6.2)	151 (6.0)		
Educational attainment				
No schooling	42 (1.7)	36 (1.4)	0.128	0.033
Primary	131 (5.3)	147 (5.9)		
Junior secondary	67 (2.7)	105 (4.2)		
Senior secondary	454 (18.2)	439 (17.6)		
Technical	361 (14.5)	368 (14.7)		
Undergraduate	1314 (52.7)	1287 (51.6)		
Graduate	100 (4.0)	92 (3.7)		
Postgraduate	26 (1.0)	21 (0.8)		
Income (CNY)				
≤2000	426 (10.7)	439 (17.5)	0.993	0.014
2001–4000	720 (28.7)	712 (28.3)		
4001–6000	601 (23.9)	599 (23.8)		
6001–12000	483 (19.2)	482 (19.2)		
≥12001	282 (11.2)	280 (11.1)		
Smoking				
Never smoked	2067 (82.3)	2020 (80.4)	0.179	0.052
Quit	75 (3.0)	92 (3.7)		
Still smoke	670 (14.7)	400 (15.9)		
Alcohol usage				
Never drink	1494 (59.5)	1477 (58.8)	0.616	0.037
Recently drink	261 (10.4)	274 (10.9)		
Quit	294 (11.7)	318 (12.7)		
Always drink	463 (18.4)	443 (17.6)		
Sleep time (hours)				
<5	675 (26.9)	700 (27.9)	0.038	0.082
5–6	929 (37.0)	892 (35.5)		
6–7	646 (25.7)	601 (23.9)		
>7	262 (10.4)	319 (12.7)		

IF, Intermittent fasting; SMD, Standard mean differences.

### Multiple logistic regression

Subsequently, we performed logistic regression analysis on multiple variables to investigate their significant impacts on PA (Table 2). We found that IF significantly was significantly associated with lower levels of PA (OR = 0.769, 95% CI: 0.657–0.900, *p* < 0.01). Similar effects were observed for individuals with BMI of 18.5–23.9 (OR = 0.768, 95% CI: 0.606–0.980, *p* < 0.05) and those with sleep duration of 6–7 h (OR = 0.796, 95% CI: 0.638–0.992, *p* < 0.05). Regarding the factor of gender (OR = 1.976, 95% CI: 1.619–2.421, *p* < 0.001), females exhibited higher levels of PA compared to males.

**TABLE 2:** Multiple logistic regression of PA (N = 5024).

Variables	PA		
	Adjusted OR	95% CI	p
Intermittent fasting			
No	1	Reference	
Yes	0.7692	(0.6567, 0.9004)	<0.01
Gender			
Male	1	Reference	
Female	1.9756	(1.6187, 2.4217)	<0.001
Age (years)			
18–34	1	Reference	
35–44	1.0858	(0.8475, 1.3809)	0.508
45–59	0.8936	(0.6824, 1.1595)	0.405
60–80	0.9477	(0.5750, 1.5025)	0.826
BMI (kg/m^2^)			
<18.5	1	Reference	
18.5–23.9	0.7682	(0.6064, 0.9803)	<0.05
24.0–27.9	0.8836	(0.6610, 1.1840)	0.405
≥28.0	1.1342	(0.7707, 1.6541)	0.517
Educational attainment			
No schooling	1	Reference	
Primary	0.7821	(0.3998, 1.6004)	0.485
Junior secondary	0.9064	(0.4393, 1.9341)	0.794
Senior secondary	0.8196	(0.4403, 1.6155)	0.546
Technical	0.9010	(0.4826, 1.7803)	0.752
Undergraduate	0.7427	(0.4039, 1.4502)	0.359
Graduate	0.4614	(0.2088, 1.0341)	0.056
Postgraduate	1.2914	(0.4923, 3.3052)	0.596
Income (CNY)			
≤2000	1	Reference	
2001–4000	0.9214	(0.7288, 1.1677)	0.496
4001–6000	0.8407	(0.6556, 1.0793)	0.172
6001–12000	0.8699	(0.6690, 1.1310)	0.298
≥12001	0.8592	(0.6264, 1.1716)	0.342
Smoking			
Never smoked	1	Reference	
Quit	0.7235	(0.4128, 1.1934)	0.230
Still smoke	0.9479	(0.7233, 1.2333)	0.694
Alcohol usage			
Never drink	1	Reference	
Recently drink	1.0471	(0.7977, 1.3599)	0.735
Quit	1.1893	(0.9194, 1.5260)	0.179
Always drink	1.1357	(0.8967, 1.4310)	0.285
Sleep time (hours)			
<5	1	Reference	
5–6	0.8352	(0.6862, 1.0169)	0.072
6–7	0.7959	(0.6377, 0.9919)	<0.05
>7	0.9842	(0.7434, 1.2942)	0.910

PA, physical activity.

### Subgroup analyses on sleep time

**FIGURE 2:**
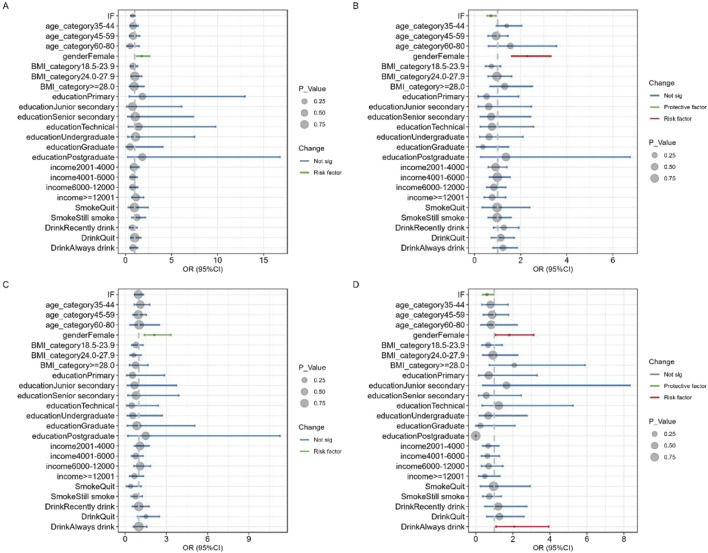
Subgroup analyses on sleep time: **(A)** < 5 h, **(B)** 5–6 h, **(C)** 6–7 h, **(D)** > 7 h.

To explore potential heterogeneity in the effects of IF across different population groups and identify potential effect modifiers that may be overlooked in the overall analysis, we conducted subgroup analyses stratified by different sleep duration, as shown in Figure 2. In the subgroups with sleep duration of 5–6 h (OR = 0.707, 95% CI: 0.539–0.924) and more than 7 h (OR = 0.601, 95% CI: 0.368–0.974), IF was observed to significantly impact PA levels, resulting in declined PA levels. Conversely, in the subgroup with sleep duration of less than 5 h (OR = 1.772, 95% CI: 1.217–2.621) and 6–7 h (OR = 2.129, 95% CI: 1.390–3.334), females exhibited lower levels of PA compared to males, while the opposite was true in the other two groups. Additionally, in the subgroup with sleep durations exceeding 7 h, individuals with drinking habits were more likely to engage in PA (OR = 2.086, 95% CI: 1.093–3.944).

### Mediation analyses on sleep time

To explore whether sleep duration mediates the association between IF and PA, a formal mediation analysis was conducted, as shown in Supplementary Figure S1. In this model, IF served as the independent variable, PA as the dependent variable, and sleep duration as the proposed mediator.

The results indicated that the average causal mediation effect (ACME) was not statistically significant (ACME = 0.0004, *p* = 0.67), suggesting that sleep duration did not significantly mediate the relationship between IF and PA. Similarly, the average direct effect (ADE) was also not statistically significant (ADE = 0.007, *p* = 0.42), and the total effect (TE = 0.007, *p* = 0.40) remained non-significant. The proportion mediated was estimated at approximately 5.8%, but this effect was also non-significant (*p* = 0.77), with wide confidence intervals.

These results suggest that while sleep duration differed between IF and non-IF groups, it did not serve as a statistically significant mediator in the pathway linking IF to PA within the current cross-sectional framework.

## Discussion

This study aimed to determine the prevalence of IF among Chinese residents aged 18–80 years and examine whether IF is associated with PA intensity. Through PSM, the study proved that participants with IF were more likely to engage in lower-intensity PA than those without IF.

### The prevalence of the IF

In this cross-sectional study of 25,679 residents who came from 23 provinces, four municipalities, and five autonomous regions in mainland China, it was found that 9.78% (2,512) of individuals aged 18–80 years adopted the IF dietary strategy with the incidence reaching 70.78% (1,778) among residents aged 18–34 years. Compared to a previous study on the youth population in Canada (Ganson et al., 2022), IF was more common among Chinese residents. IF is a convenient dietary method that can help control weight, and reduce blood sugar and cholesterol levels and is favoured by an increasing number of residents. The high prevalence of IF in China may also be attributed to the sociological characteristics of different populations (Liu et al., 2015). The IF diet may be more easily embraced by young people, especially young women, who are more likely to be exposed to social media and stress body image (Lowe et al., 2020). In the sample of this study, the proportion of people aged 18–34 is the highest among different age groups, and the proportion of women is higher than that of men. Previous studies have demonstrated that women often engage in different types of PA compared to men, favoring more structured or aerobic activities (Regina et al., 2018). This trend may be driven by a greater emphasis on maintaining cardiovascular health or managing body composition. As such, the gender differences observed in this study are consistent with broader patterns of PA behavior seen in the research, warranting further investigation into the underlying mechanisms. So it may to some extent lead to a higher prevalence of IF in the results of this study.

### Factors associated with PA

The most important finding of this study is the association between IF and lower levels of PA, which can be attributed to both physiological and psychological factors. However, it is important to emphasize that this relationship is correlational and does not imply causation due to the cross-sectional nature of the study. Typically, low-intensity PA do not require substantial energy support, so individuals engaging in such activities may experience increased fatigue or incapacity during IF periods (Rizzoli et al., 2021). Moreover, although previous studies have indicated that IF can positively impact cardiovascular health indicators, growth hormone production, and muscle growth—including improvements in hyperandrogenism and menstrual status in women (Currenti et al., 2021)—insufficient energy may prevent participants from effectively performing daily light exercises, potentially reducing their quality of life (Ge et al., 2019).

In addition to physical effects, IF leading to lower levels of PA may also be influenced by psychological factors. Psychologically, IF may cause individuals accustomed to inactive PA to focus excessively on food, thereby increasing their psychological burden. Research has shown that IF can alter an individual’s appetite and dietary preferences, which for those used to light exercise, may result in uneven nutrient intake, subsequently affecting mood and cognitive function (Asher and Schibler, 2011). Moreover, IF may intensify feelings of hunger and decrease both the motivation and frequency of engagement in low-intensity PA (James et al., 2018). However, as this study did not incorporate standardized psychological measurements, the hypothesized psychological mechanisms should be interpreted with caution and further verified in future research using validated scales.

Thus, although IF can be beneficial for health in some instances, it must be implemented carefully, considering individual activity needs and lifestyles. For individuals who habitually engage in low levels of PA, personalized guidance is necessary to ensure a balance between energy intake and expenditure, maintaining their daily activity requirements. It is also crucial to enhance awareness of mental health importance, especially among young people who may be more vulnerable to body image disorders and might adopt extreme weight loss measures. Individuals should be mindful of the potential stress caused by IF to their mental health and avoid neglecting their feelings or over-exercising (Rubén et al., 2023). Integrating warm-up and relaxation exercises before and after PA, along with low-intensity activities beneficial for both body and mind, such as yoga and meditation, enhances overall health (Simon et al., 2021). This approach can facilitate the sustained maintenance of both IF and PA over time and mitigate any associated risks (Edlund et al., 2022).

Although a significant difference in sleep duration was observed between IF and non-IF groups, mediation analysis in this study did not support sleep duration as a significant mediator between IF and PA. It is important to consider the potential confounding effects in the observed association between IF, sleep duration, and PA. Factors such as psychological stress, dietary intake, or work-related physical exhaustion could also influence both sleep and PA levels. Future research should control for these variables to better isolate the effects of IF on sleep and PA.

While the strict caliper value (0.001) optimized covariate balance, it reduced the matched sample size by 83.5% (from 30,503 to 5,024 participants). This reduction may diminish statistical power; however, *post hoc* power analysis indicated 85% power to detect the observed OR = 0.77 (α = 0.05). Nevertheless, generalizability may be constrained to populations with characteristics similar to the matched cohort. Future studies should employ adaptive matching strategies to balance precision and representativeness.

This study also explored the relationship between PA levels and various variables. Interestingly, findings suggest that both abstainers and those with current or recent alcohol consumption exhibit higher levels of PA compared to individuals who have never consumed alcohol. This highlights the complex interplay between self-control, health literacy, and PA levels (Rizzoli et al., 2021).

## Limitations

The study has several limitations that should be acknowledged. Firstly, it is a cross-sectional survey, it is challenging to establish a causal relationship between IF and PA levels. Additionally, the PSM method has certain limitations to consider as well (Shi et al., 2022). Although PSM helps to balance observed covariates, it cannot account for unmeasured confounders such as psychological state, total energy intake, or motivation for behavior change. Future studies should consider stratified analyses based on BMI categories and total caloric intake to assess whether IF affects PA differently across weight groups or dietary patterns. The use of a narrow caliper (0.001) prioritized internal validity at the potential cost of external validity.

Moreover, extra limitations include the potential for self-report bias in the data collection process, as both IF and PA levels were self-reported, which could lead to over- or under-estimation of these behaviors. Furthermore, the measurement of IF was relatively simplistic, focusing on only a few basic parameters without accounting for variations in dietary intake or fasting duration. Lastly, there remains the possibility of residual confounding due to unmeasured variables such as participants’ overall dietary energy intake or psychological stress levels, which may have influenced the results.

## Conclusion

Intermittent fasting was found to be relatively common among Chinese residents aged 18–80 years, with a higher prevalence observed in the 18–34 years age group. The intensity of PA varied significantly between residents with and without IF behaviour. While a significant association was observed, the cross-sectional nature of the study does not allow for causal inference. Specifically, residents with IF behaviour were more likely to engage in low-intensity PA, but there were no mediation effects of sleep duration between IF and PA. Future research should further investigate this tripartite relationship between IF, PA and sleep, using longitudinal or experimental designs and incorporating validated psychological assessments. These efforts should focus on identifying the optimal fasting windows and dietary combinations that support individual sleep patterns and PA to enhance overall wellbeing and promote personalized guidance based on individual behavioral and health profiles. To enhance the sustainability and safety of residents’ dietary and exercise routines it is crucial to provide scientific health education to the public.

## Data Availability

The raw data supporting the conclusions of this article will be made available by the authors, without undue reservation.

## Abbreviations

IF, Intermittent fasting; PA, Physical activity; IPAQ, International Physical Activity Scale; BMI, Body Mass Index; PSM, Propensity Score Matching; PBICR, Psychology and Behaviour Investigation of Chinese Residents; SMD, Standard mean differences; OR, Odds Ratio; CI, Confidence Interval; MET, Metabolic Equivalent.
