# Corrigendum: Impact of intermittent fasting on physical activity: a national survey of Chinese residents aged 18–80 years

**DOI:** 10.3389/fphys.2025.1628669

**Published:** 2025-05-30

**Authors:** Feiying He, Shiyu Bai, Xiangchun Xu, Jingqiao Miao, Hongwen Yu, Jiale Qiu, Yibo Wu, Yangdong Fan, Lei Shi

**Affiliations:** ^1^ School of Health Management, Southern Medical University, Guangzhou, Guangdong, China; ^2^ School of Basic Medical Sciences, Southern Medical University, Guangzhou, Guangdong, China; ^3^ Guangdong Provincial People’s Hospital (Guangdong Academy of Medical Sciences), Southern Medical University, Guangzhou, Guangdong, China; ^4^ School of Public Health, Southern Medical University, Guangzhou, Guangdong, China; ^5^ School of Stomatology, Southern Medical University, Guangzhou, Guangdong, China; ^6^ School of Public Health, Peking University, Beijing, China; ^7^ School of Health Management, Guangzhou Medical University, Guangzhou, Guangdong, China

**Keywords:** intermittent fasting, physical activity, multiple logistic regression, health management, China

In the published article, there was an error in “Figure 1 Two levels of physical activity” as published. In this figure, there was an error in the box “No,Inactive (PA = 0)”, the correct form should be “No, Inactive (PA = 1).”

The corrected Figure 1 and its caption appear below.

**FIGURE 1:**
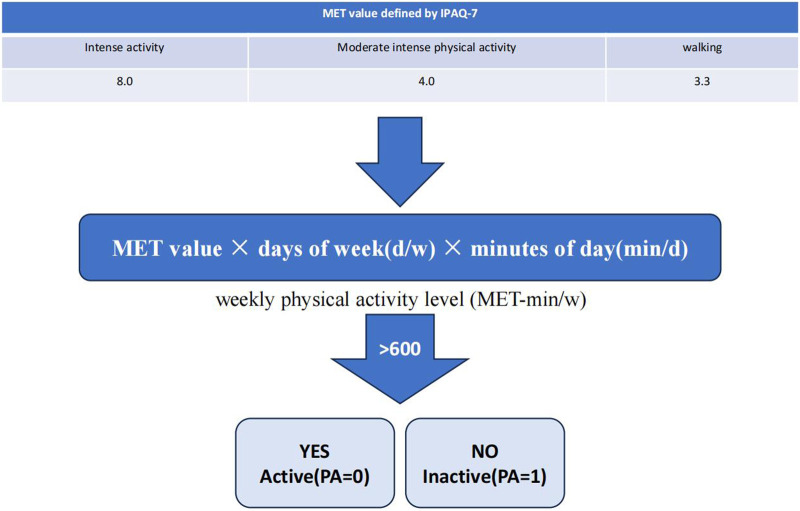
Two levels of physical activity.

The authors apologize for this error and state that this does not change the scientific conclusions of the article in any way. The original article has been updated.

