# Supplementary material for: Impact of intermittent fasting on physical activity: a national survey of Chinese residents aged 18–80 years
Source: Front Physiol. 2025 May 12;16:1582036. doi: 10.3389/fphys.2025.1582036 (PMC12105048; doi:10.3389/fphys.2025.1582036)
Supplement: Supplementary file 1 [file DataSheet1.pdf]

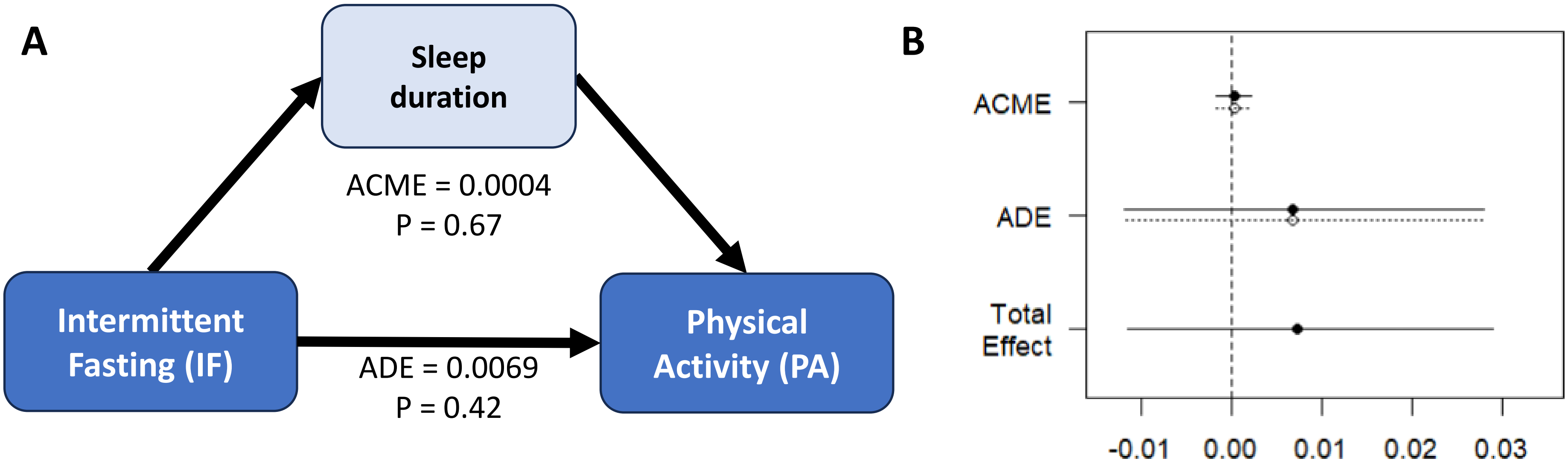

Supplementary Figure 1. Mediation analysis of the relationship between intermittent fasting (IF), sleep duration, and physical activity (PA).

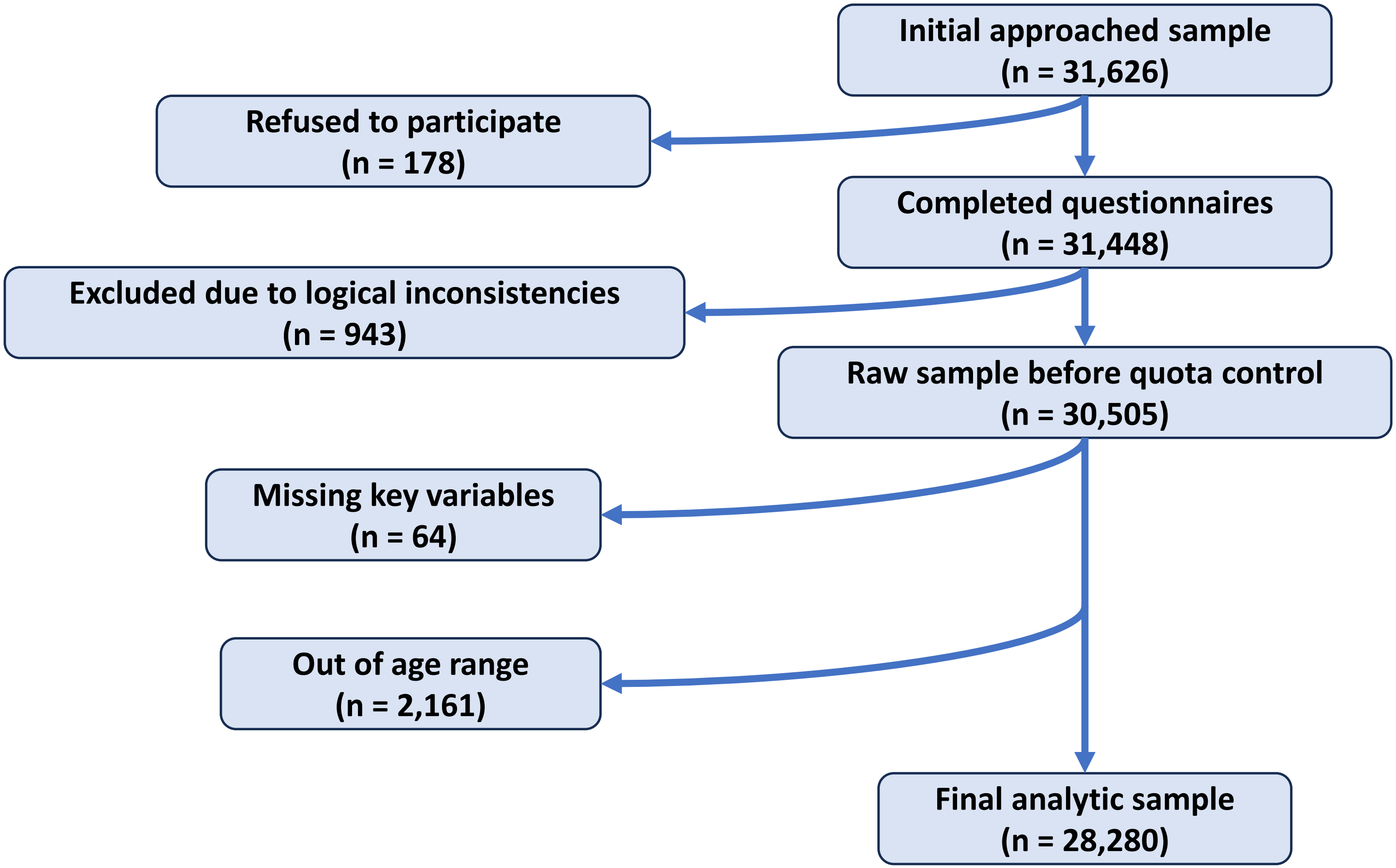

Supplementary Figure 2. Flow diagram of participant recruitment and sample selection.
